# Organochlorine Pesticides and Risk of Endometriosis: Findings from a Population-Based Case–Control Study

**DOI:** 10.1289/ehp.1306648

**Published:** 2013-11-05

**Authors:** Kristen Upson, Anneclaire J. De Roos, Mary Lou Thompson, Sheela Sathyanarayana, Delia Scholes, Dana Boyd Barr, Victoria L. Holt

**Affiliations:** 1 Department of Epidemiology, School of Public Health, University of Washington, Seattle, Washington, USA; 2 Division of Public Health Sciences, Fred Hutchinson Cancer Research Center, Seattle, Washington, USA; 3 Department of Biostatistics, and; 4 Department of Environmental and Occupational Health Sciences, School of Public Health, University of Washington, Seattle, Washington, USA; 5 Department of Pediatrics, School of Medicine, University of Washington, Seattle, Washington, USA; 6 Seattle Children’s Research Institute, Seattle, Washington, USA; 7 Group Health Research Institute, Seattle, Washington, USA; 8 Emory University, Rollins School of Public Health, Atlanta, Georgia, USA

## Abstract

Background: Endometriosis is considered an estrogen-dependent disease. Persistent environmental chemicals that exhibit hormonal properties, such as organochlorine pesticides (OCPs), may affect endometriosis risk.

Objective: We investigated endometriosis risk in relation to environmental exposure to OCPs.

Methods: We conducted the present analyses using data from the Women’s Risk of Endometriosis (WREN) study, a population-based case–control study of endometriosis conducted among 18- to 49-year-old female enrollees of a large health care system in western Washington State. OCP concentrations were measured in sera from surgically confirmed endometriosis cases (*n* = 248) first diagnosed between 1996 and 2001 and from population-based controls (*n* = 538). We estimated odds ratios (OR) and 95% CIs using unconditional logistic regression, adjusting for age, reference date year, serum lipids, education, race/ethnicity, smoking, and alcohol intake.

Results: Our data suggested increased endometriosis risk associated with serum concentrations of β-hexachlorocyclohexane (HCH) (third vs. lowest quartile: OR = 1.7; 95% CI: 1.0, 2.8; highest vs. lowest quartile OR = 1.3; 95% CI: 0.8, 2.4) and mirex (highest vs. lowest category: OR = 1.5; 95% CI: 1.0, 2.2). The association between serum β-HCH concentrations and endometriosis was stronger in analyses restricting cases to those with ovarian endometriosis (third vs. lowest quartile: OR = 2.5; 95% CI: 1.5, 5.2; highest vs. lowest quartile: OR = 2.5; 95% CI: 1.1, 5.3).

Conclusions: In our case–control study of women enrolled in a large health care system in the U.S. Pacific Northwest, serum concentrations of β-HCH and mirex were positively associated with endometriosis. Extensive past use of environmentally persistent OCPs in the United States or present use in other countries may affect the health of reproductive-age women.

Citation: Upson K, De Roos AJ, Thompson ML, Sathyanarayana S, Scholes D, Barr DB, Holt VL. 2013. Organochlorine pesticides and risk of endometriosis: findings from a population-based case–control study. Environ Health Perspect 121:1319–1324; http://dx.doi.org/10.1289/ehp.1306648

## Introduction

Endometriosis, characterized by the presence of endometrial glands and stroma outside of the uterus, is associated with substantial morbidity, including severe, chronic pelvic pain, heavy menstrual bleeding, and infertility (Eskenazi and Warner 1997; Nisolle and Donnez 1997). This serious, chronic condition is estimated to affect 6–10% of reproductive-age women in the United States (Eskenazi and Warner 1997). Although its etiology is not fully understood, endometriosis is considered an estrogen-dependent disease. Endometriosis rarely is seen before the onset of menses or after menopause (Houston 1984), and suppression of ovarian hormone production—as with combined oral contraceptives, progestins, and gonadotropin-releasing hormone agonist therapy—reduces symptoms associated with the condition such as chronic pelvic pain and heavy menstrual bleeding (Giudice 2010). Investigations into the pathophysiology of endometriosis suggest that disease onset and progression involve steroid-related alterations of the endometrium and peritoneal cavity, excess estrogen production by ectopic endometriotic lesions, and changes in ovarian steroidogenesis (Bulun 2009; Giudice and Kao 2004; Ulukus et al. 2006). Thus, environmental chemicals that are endocrine disruptive, or that mimic or alter endogenous hormonal activity, may plausibly affect endometriosis risk.

Organochlorine pesticides (OCPs) are synthetic pesticides that were widely used in the latter half of the 20th century. Despite bans and restrictions on OCP use in the United States over the past several decades, the U.S. population still has detectable serum concentrations of OCPs because of the environmental persistence of the chemicals and their bioaccumulation within organisms and up the food chain [Centers for Disease Control and Prevention (CDC) 2009]. Currently, U.S. general population exposure to OCPs occurs primarily through consumption of contaminated fatty foods, fish, and dairy products (CDC 2009; Patterson et al. 2009). Additionally, continued global use and unintentional production of these chemicals may contribute to ongoing U.S. population exposure given that these chemicals have the capacity for long-range transport, appearing in locations far from where they are manufactured or used (Fisher 1999). Continuing OCP exposure is of potential human health concern because these chemicals generally have demonstrated estrogenic properties in *in vitro* studies (Andersen et al. 2002; Shelby et al. 1996; Soto et al. 1995) and have exhibited adverse reproductive system effects in laboratory animal studies, altering uterine and ovarian function and endogenous hormone production (Alvarez et al. 2000; Foster et al. 1995; Shelby et al. 1996; Van Velsen et al. 1986).

Despite evidence of endocrine-disruptive properties of OCPs, the impact of these chemicals on endometriosis risk remains unclear; prior epidemiologic studies investigating serum concentrations of OCPs in relation to endometriosis have had conflicting results (Cooney et al. 2010; Lebel et al. 1998; Niskar et al. 2009; Porpora et al. 2009; Quaranta et al. 2006; Tsukino et al. 2005). These studies were conducted primarily among women undergoing surgical evaluation by laparoscopy. Although studies restricted to women undergoing laparoscopy are generally convenient and economical and have the advantage of confirming the absence of disease among controls, these studies may have produced biased results if the indication for such evaluation was associated with OCP body burden. Only one published study has used a population-based sampling framework to evaluate endometriosis risk in relation to serum OCP concentrations, but that study cohort was limited by its small size (*n* = 127) (Buck Louis et al. 2012). The purpose of the present analyses was to use data from a large, general population-based case–control study of endometriosis to investigate, outside the specialized setting of women undergoing laparoscopy, the risk of incident, surgically confirmed endometriosis in relation to environmental exposure to OCPs.

## Materials and Methods

*Study population*. We conducted the present analyses using data from the Women’s Risk of Endometriosis (WREN) study and the ancillary Persistent Organic Pollutants and Endometriosis Risk (POPs) study. WREN was a population-based case–control study of endometriosis conducted among 18- to 49-year-old female enrollees of Group Health, a large integrated health care system in western Washington State. As previously described, cases in the WREN study were 340 women with incident first-time endometriosis diagnoses [*International Classification of Disease, 9th Revision* (ICD-9) (World Health Organization 1977) diagnostic codes 617.0–617.5, 617.8–617.9, excluding those with adenomyosis coded as 617.0, uterine endometriosis] between 1 April 1996 and 31 March 2001 (Marino et al. 2008; Trabert et al. 2010). The diagnoses were confirmed by record review indicating the presence of endometriosis through direct surgical visualization, with histologic confirmation when available. The reference date for cases was the date of first visit to Group Health for symptoms leading to endometriosis diagnosis. Population-based controls (*n* = 741) in the WREN study were female Group Health enrollees without diagnosed endometriosis, randomly selected from computerized Group Health enrollment databases and frequency matched to cases in 5-year age groups. Controls were assigned reference dates to correspond with the distribution of reference dates among cases. Cases and controls without a uterus, at least one ovary, or 6 months minimum enrollment at reference date were excluded, as were menopausal or postmenopausal women. Because we were interested in first diagnosis of endometriosis, we also excluded women with a past history of the disease. WREN subjects participated in a structured, in-person interview covering a range of topics including reproductive history and contraceptive use as well as medical and family history and lifestyle behaviors before the reference date. The cases and controls who participated in WREN and completed the interview represented 73% of those invited to participate (Marino et al. 2008). In the POPs study, a subset of WREN study participants was invited to donate a blood sample to assess exposure to OCPs and polychlorinated biphenyls (PCBs); 89.7% of these cases (*n* = 286) and 85.1% of these controls (*n* = 592) agreed, and 283 cases and 585 controls donated samples.

Quantification of serum OCP concentrations was completed for 268 cases and 550 controls. Given information collected during the WREN study interview, we discovered a past history of surgically confirmed endometriosis for 20 participants (8 cases and 12 controls), whom we then excluded. We also excluded 11 cases not meeting the definition of definite or possible endometriotic disease (Holt and Weiss 2000), a definition that focuses on endometriosis with evidence of tissue invasiveness or interference with normal physiologic processes. One case whose endometriosis diagnosis was not confirmed surgically also was excluded. Thus, in this analysis we used data from 248 cases and 538 controls in the WREN study. The Fred Hutchinson Cancer Research Center Institutional Review Board approved this study, and each participant provided written informed consent before enrollment and participation.

*Serum organochlorine pesticide measurements*. Study personnel collected nonfasting blood samples at the time of interview. The blood was processed by the Fred Hutchinson Cancer Research Center Specimen Processing Laboratory, and serum was aliquoted into acid-washed glass vials and stored at –20°C before shipment to the Toxicology Branch, Division of Laboratory Sciences, National Center for Environmental Health, CDC (Atlanta, GA) for measurement of serum OCP concentrations. OCP analytes were isolated from serum using solid-phase extraction and quantified using isotope-dilution gas chromatography–high resolution mass spectrometry (Barr et al. 2003, 2006). The 11 OCPs or metabolites quantified were β-hexachlorocyclohexane (β-HCH), γ-hexachlorocyclohexane (γ-HCH), heptachlor epoxide, oxychlordane, *trans*-nonachlor, two isomers of dichlorodiphenyltrichloroethane (*p,p´*-DDT, *o,p´*-DDT), dichlorodiphenyldichloroethylene (*p,p´*-DDE), dieldrin, hexachlorobenzene, and mirex. The limit of detection (LOD) for all OCPs was 10.0 pg/g serum with relative SDs of < 15%. The laboratory staff was blinded to the case status of the laboratory specimens.

Quality control (QC) procedures monitored the intrabatch variability in serum OCP measurements with 34 QC duplicate samples. The agreement between measurements for duplicate samples included in the same analytic run was high, with intraclass correlation coefficients ranging from 89% to 99% for individual OCP analytes.

Laboratory personnel quantified the individual lipid components total cholesterol, free cholesterol, triglycerides, and phospholipids in each serum sample using enzymatic methods (Roche Chemicals, Indianapolis, IN) (Phillips et al. 1989). We then determined serum total lipid concentrations (milligrams per deciliter) by summing the individual lipid components using an established formula: total lipids = 1.677 × (total cholesterol – free cholesterol) + free cholesterol + triglycerides + phospholipids (Akins et al. 1989). A shorter formula, [total lipids = (2.27 × total cholesterol) + triglycerides + 62.3], was used if free cholesterol was not quantified (9.7% cases, 10.4% controls) (Akins et al. 1989; Bernert et al. 2007; Phillips et al. 1989).

*Exposure coding*. Serum OCP measurements that were not reportable due to interference with co-eluting chemicals were excluded (Table 1). We created categorical variables for each serum OCP analyte; values < LOD were always included in the lowest category. For analytes with ≤ 25% < LOD, we categorized the serum OCP concentration by quartiles of the control distribution. We similarly categorized the serum concentration of dieldrin, an OCP with slightly more than 25% of values < LOD, but we used the LOD value (10.0 pg/g serum) as the first quartile cutpoint. For two OCPs with > 25% of values < LOD (mirex and γ-HCH), we categorized serum OCP concentrations into three categories: ≤ LOD, less than or equal to, and greater than the median value among controls with detectable concentrations. By summing the molar concentrations of the individual analytes within each group, we also created three summary exposure variables among structurally related or isomeric forms of OCPs: Σchlordane (sum of oxychlordane, heptachlor epoxide, and *trans*-nonachlor), ΣDDT (sum of *p,p´*-DDT and *p,p´*-DDE), and ΣHCH (sum of β-HCH and γ-HCH). The summary exposure variables were categorized by quartiles based on the distribution in controls.

**Table 1:** Laboratory measurement of serum organochlorine pesticides and distribution by case status, Group Health, 1996–2001 (*n* = 786).

Pesticide (pg/g serum)	Measured ≥ LOD [*n* (%)]	Measured < LOD [*n* (%)]	Interference [*n* (%)]	Cases (*n *= 248) [median (IQR)]	Controls (*n *= 538) [median (IQR)]
β-HCH	709 (90.2)	30 (3.8)	47 (6.0)	51.91 (29.19, 80.79)	43.06 (26.99, 74.03)
γ-HCH^*a*^	339 (43.1)	424 (53.9)	23 (2.9)	< 10.00 (< 10.00, 13.97)	< 10.00 (< 10.00, 13.34)
Heptachlor epoxide	542 (69.0)	102 (13.0)	142 (18.1)	27.52 (18.88, 42.89)	26.19 (16.93, 42.91)
Oxychlordane	613 (78.0)	114 (14.5)	59 (7.5)	60.59 (30.93, 94.78)	51.96 (28.31, 79.55)
*trans*-Nonachlor	784 (99.7)	2 (0.3)	0 (0.0)	81.22 (53.76, 127.63)	75.15 (51.37, 107.65)
*p,p′*-DDE	784 (99.7)	0 (0.0)	2 (0.3)	1569.74 (947.52, 2799.01)	1575.51 (905.86, 2816.55)
*p,p′*-DDT	670 (85.2)	80 (10.2)	36 (4.6)	29.11 (19.03, 45.10)	28.36 (18.79, 44.38)
Dieldrin	513 (65.3)	210 (26.7)	63 (8.0)	48.79 (< 10.00, 71.40)	48.18 (< 10.00, 73.74)
Hexachlorobenzene	786 (100)	0 (0.0)	0 (0.0)	287.87 (177.03, 529.99)	279.00 (159.74, 589.30)
Mirex^*a*^	300 (38.2)	453 (57.6)	33 (4.2)	< 10.00 (< 10.00, 15.61)	< 10.00 (< 10.00, 13.11)
IQR, interquartile range. ^***a***^For categorizing exposures in the statistical analyses, median and IQR calculated among controls using values ≥ LOD. For γ-HCH, median (IQR) was 13.89 (12.21, 17.65). For mirex, median (IQR) was 15.47 (12.12, 23.32).					

We imputed serum measurements quantified as < LOD using a distribution-based multiple imputation procedure, to be able to create the summary exposure variables and to conduct the test of trend analyses (Lubin et al. 2004). Hence, we used imputed values only in analyses requiring continuous OCP analyte concentrations. This procedure entailed creating a bootstrap sample of the data and estimating the parameters of the log-normal serum OCP distribution among controls by maximum likelihood estimation, including the covariates age, reference year, smoking, alcohol, education, race/ethnicity, natural log-transformed total lipids, breastfeeding, and body mass index (BMI). The log-normal distribution with the estimated parameters was then randomly sampled to impute values < LOD. The process was repeated to create five multiple imputed data sets. Given that this imputation procedure has demonstrated minimal bias with 50–60% missing values < LOD in a simulation study (Lubin et al. 2004), all analytes detected in ≥ 30% of samples were included in analyses (Table 1). The OCP analyte *o,p´*-DDT was not included in statistical analyses as it was detected in only 8.8% of samples.

*Statistical analyses*. The statistical analyses were conducted using STATA version 12.0 (StataCorp, College Station, TX) and SAS version 9.3 (SAS Institute Inc., Cary, NC). We used the significance level of α = 0.05 in all analyses. We summarized the distribution of OCPs using the median and interquartile range. To evaluate the degree of collinearity among pairs of OCP analytes, we conducted pairwise Spearman correlation using the non-imputed continuous serum OCP concentration data restricted to values ≥ LOD.

We used unconditional logistic regression to estimate odds ratios (ORs) and 95% CIs for associations between serum concentrations of OCPs and endometriosis. The categories for each OCP analyte or summary metric were modeled as a set of indicator variables, with the lowest category serving as the reference category. We identified variables necessary for adjustment in the model using a directed acyclic graph (DAG), informed by previous studies of endometriosis risk factors and predictors of serum OCP concentrations (Greenland et al. 1999; Hernan et al. 2002). On this basis we adjusted for natural logarithm-transformed total serum lipids (continuous), education (≤ high school graduate, some college, college graduate/post graduate), race/ethnicity (non-Hispanic white, non-Hispanic black, non-Hispanic Asian/Pacific Islander, other/Hispanic), smoking (never, former, current), alcohol intake (never, former, current), and the frequency matching variables age at reference date (≤ 19, 20–24, 25–29, 30–34, 35–39, 40–44, 45–49 years) and reference date year (1995, 1996, 1997, 1998, 1999, 2000, 2001). We did not adjust for parity because it may be an intermediate in the causal pathway between exposure and development of endometriosis, or may be affected by endometriosis (Koepsell and Weiss 2003). Additionally, we did not adjust for other OCPs and PCBs because of the concern for collinearity, with resulting unstable coefficient estimates or lack of model convergence when estimating multiple correlated exposure effects with maximum likelihood estimation. We instead conducted a conventional one-at-a-time analysis, considering each individual categorical OCP analyte or summary exposure variable in a separate logistic regression model. We also repeated the analyses restricting cases to those with ovarian endometriosis (*n* = 132), which may be etiologically distinct from non-ovarian disease (Nisolle and Donnez 1997).

To test the trend across categories of an individual serum OCP analyte or summary exposure variable, we created a continuous variable assigning the median category values to participants in each category and included the variable in the adjusted logistic regression model. We interpreted the *p*-value accompanying the continuous variable for the test of trend.

In logistic regression analyses using imputed data, namely analyses of summary exposure variables and tests of trend, we used the PROC MIANALYZE procedure in SAS to combine results from individual imputed data sets to account for uncertainty in the imputation and adjust the variance of the estimates.

We conducted two exploratory analyses. The first was based on our hypothesis that body burden of OCPs may be associated with other hormonally related conditions or infertility leading to laparoscopic evaluation. Thus, controls restricted to women undergoing laparoscopy or reproductive assistance may have atypical serum OCP concentrations. To investigate this hypothesis, we used the Wilcoxon rank sum test to explore differences in the distribution of serum OCPs concentrations between controls with and without a history of laparoscopy and between controls with and without a history of infertility testing. In the second exploratory analysis, we considered an alternative conceptual framework with factors of parity and breastfeeding as confounders, because excretion pathways of lactation and parturition as well as physiologic volume changes in pregnancy may reduce OCP body burden (Verner et al. 2008). In this analysis we adjusted for parity and breastfeeding using a composite variable (nulliparous women, parous women with lifetime history of breastfeeding ≤ 6 months, parous women with lifetime history of breastfeeding > 6 months).

## Results

We found that cases and controls were similar demographically, except that cases were more likely to be of Hispanic ethnicity (Table 2). Additionally, cases were more likely than controls to be current alcohol consumers and nulliparous, less likely to have a history of breastfeeding for > 1 year, and had greater serum lipid concentrations. The distribution of characteristics in this subset of WREN participants was similar to that found among WREN cases and controls in the parent study (data not shown). The demographic and lifestyle characteristics among controls of the WREN study closely mirror those of the general population in the surrounding Puget Sound region (Saunders et al. 2005)

**Table 2:** Characteristics of WREN participants with serum organochlorine pesticide measurements, Group Health, 1996–2001 [*n* (%)].

Characteristic	Cases (*n *= 248)	Controls (*n *= 538)
Age (years)
17–24	19 (7.7)	44 (8.2)
25–34	52 (21.0)	93 (17.3)
35–44	120 (48.4)	277 (51.5)
45–49	57 (23.0)	124 (23.1)
Race
White	213 (85.9)	457 (85.1)
Black	8 (3.2)	22 (4.1)
Asian/Pacific Islander	20 (8.1)	38 (7.1)
American Indian/Aleut/Eskimo	2 (0.8)	4 (0.7)
More than one race	5 (2.0)	16 (3.0)
Ethnicity
Hispanic	16 (6.5)	18 (3.4)
Non-Hispanic	230 (93.5)	518 (96.6)
Income
< $35,000	73 (30.5)	146 (28.0)
$35,000–$69,999	105 (43.9)	223 (42.7)
≥ $70,000	61 (25.5)	153 (29.3)
Education
< High school	8 (3.2)	17 (3.2)
High school graduate	42 (16.9)	96 (17.8)
Some college	80 (32.3)	202 (37.6)
College graduate	79 (31.9)	120 (22.3)
Postgraduate	39 (15.7)	103 (19.1)
Cigarette smoking
Never	143 (57.7)	325 (60.4)
Former	56 (22.6)	124 (23.1)
Current	49 (19.8)	89 (16.5)
Alcohol use
Never	68 (27.5)	186 (34.6)
Former	45 (18.2)	113 (21.0)
Current	134 (54.3)	239 (44.4)
BMI (kg/m^2^)
< 18.5	8 (3.2)	11 (2.1)
18.5–< 25.0	126 (50.8)	278 (52.1)
25.0–< 30.0	60 (24.2)	140 (26.2)
≥ 30.0	54 (21.8)	105 (19.7)
Parity
Nulliparous	121 (48.8)	157 (29.2)
Parous	127 (51.2)	380 (70.8)
Lifetime lactation history (weeks)
Did not breastfeed, nulliparous	121 (48.8)	157 (29.2)
Did not breastfeed, parous	27 (10.9)	71 (13.2)
1–24 weeks	42 (16.9)	89 (16.6)
25–52 weeks	28 (11.3)	77 (14.3)
> 52 weeks	30 (12.1)	143 (26.6)
Total lipids (mg/dL) [median (IQR)]	679.7 (593.4, 816.1)	661.4 (572.6, 777.1)
IQR, interquartile range.		

The distributions of serum OCP concentrations were right-skewed (Table 1). Geometric mean lipid-adjusted serum concentrations of β-HCH, oxychlordane, *trans*-nonachlor, and *p,p´*-DDE were somewhat lower than those reported by the CDC for the same years using data from the National Health and Nutrition Examination Survey (NHANES) on females ≥ 12 years of age (CDC 2009) (data not shown). The pairwise Spearman correlations among serum OCP concentrations were ≥ 0.70 for β-HCH and oxyclordane (*r* = 0.73), heptachlor epoxide and dieldrin (*r* = 0.78), and oxychlordane and *trans-*nonachlor (*r* = 0.90). Blood was collected 6 months to 5.8 years after diagnosis date in cases (median, 1.2 years).

Our data suggested increased risks of endometriosis in association with serum concentrations of β-HCH (third vs. lowest quartile: OR = 1.7; 95% CI: 1.0, 2.8, *p* = 0.047; highest vs. lowest quartile: OR = 1.3; 95% CI: 0.8, 2.4) and mirex (highest vs. lowest category: OR = 1.5; 95% CI: 1.0, 2.2, *p* = 0.065), adjusted for natural logarithm-transformed total serum lipids, education, race/ethnicity, smoking, alcohol intake, age, and reference date year (Table 3). We also found modest associations (OR = 1.4) between endometriosis and various quartiles of serum concentrations of ΣHCH, heptachlor epoxide, *trans*-nonachlor, Σchlordane, and hexachlorobenzene, although the confidence intervals for these associations included the null. None of the tests of trend across exposure categories was significant (*p* > 0.05) (data not shown). The association between serum concentrations of β-HCH and endometriosis was stronger in analyses restricting cases to those with ovarian endometriosis (third vs. lowest quartile: OR = 2.5; 95% CI: 1.2, 5.2; highest vs. lowest quartile: OR = 2.5; 95% CI: 1.1, 5.3; *p* = 0.023 for test of trend), a finding not replicated for mirex (Table 3).

**Table 3:** Adjusted odds ratios (aORs) and 95% CIs for the relationship between individual organochlorine pesticides and endometriosis, Group Health, 1996–2001.

Pesticide quartiles (pg/g serum)	All endometriosis		Ovarian endometriosis	
	Case/control (*n*)	aOR^*a*^ (95% CI)	Case/control (*n*)	aOR^*a*^ (95% CI)
β-HCH
≤ 26.99	49/126	1.0	17/126	1.0
> 26.99–43.06	41/127	0.8 (0.5, 1.4)	21/127	1.2 (0.5, 2.4)
> 43.06–74.01	78/127	1.7 (1.0, 2.8)	42/127	2.5 (1.2, 5.2)
> 74.01	64/127	1.3 (0.8, 2.4)	42/127	2.5 (1.1, 5.3)
γ-HCH^*b*^
≤ 10.0 (LOD)	129/295	1.0	69/295	1.0
> 10.0–13.89	54/112	1.1 (0.8, 1.7)	29/112	1.1 (0.7, 1.9)
> 13.89	61/112	1.3 (0.9, 1.9)	32/112	1.2 (0.7, 2.0)
∑HCH (mol/g serum)^*c*^
≤ 0.12	54/134	1.0	24/134	1.0
> 0.12–0.18	48/135	0.8 (0.5, 1.3)	21/135	0.8 (0.4, 1.7)
> 0.18–0.29	79/134	1.4 (0.9, 2.2)	45/134	1.8 (1.0, 3.3)
> 0.29	67/135	1.1 (0.6, 1.8)	42/135	1.6 (0.8, 3.0)
Heptachlor epoxide
≤ 17.10	40/110	1.0	21/110	1.0
> 17.10–26.19	55/110	1.4 (0.8, 2.4)	22/110	1.0 (0.5, 2.0)
> 26.19–42.91	59/110	1.4 (0.8, 2.3)	34/110	1.3 (0.7, 2.6)
> 42.91	51/109	1.2 (0.7, 2.1)	28/109	1.1 (0.5, 2.2)
Oxychlordane
≤ 28.31	52/124	1.0	24/124	1.0
> 28.31–51.96	46/125	0.8 (0.5, 1.3)	23/125	0.8 (0.4, 1.6)
> 51.96–79.55	57/126	1.0 (0.6, 1.6)	34/126	1.2 (0.6, 2.4)
> 79.55	73/124	1.2 (0.7, 2.1)	45/124	1.5 (0.8, 3.0)
*trans*-Nonachlor
≤ 51.37	55/134	1.0	21/134	1.0
> 51.37–75.15	55/135	1.0 (0.6, 1.6)	31/135	1.4 (0.7, 2.7)
> 75.15–107.65	56/134	1.0 (0.6, 1.6)	33/134	1.3 (0.7, 2.6)
> 107.65	82/135	1.4 (0.8, 2.4)	47/135	1.8 (0.9, 3.7)
∑Chlordane (mol/g serum)^*c*^
≤ 0.21	53/134	1.0	23/134	1.0
> 0.21–0.33	56/135	1.0 (0.6, 1.7)	24/135	1.0 (0.5, 1.9)
> 0.33–0.52	57/134	1.1 (0.6, 1.8)	36/134	1.5 (0.8, 2.9)
> 0.52	82/135	1.4 (0.8, 2.5)	49/135	1.8 (0.9, 3.7)
*p,p′*-DDE
≤ 905.86	55/134	1.0	25/134	1.0
> 905.86–1575.51	69/134	1.2 (0.8, 2.0)	38/134	1.4 (0.8, 2.6)
> 1575.51–2816.55	62/134	1.1 (0.7, 1.7)	31/134	1.1 (0.6, 2.1)
> 2816.55	61/135	1.0 (0.6, 1.7)	37/135	1.2 (0.6, 2.4)
*p,p′*-DDT
≤ 18.79	58/128	1.0	21/128	1.0
> 18.79–28.36	58/128	0.9 (0.6, 1.5)	33/128	1.4 (0.7, 2.6)
> 28.36–44.38	55/128	0.8 (0.5, 1.4)	32/128	1.2 (0.6, 2.3)
> 44.38	66/129	0.9 (0.6, 1.5)	38/129	1.3 (0.7, 2.6)
∑DDT (mol/g serum)^*c*^
≤ 2.88	55/135	1.0	25/135	1.0
> 2.88–5.03	71/134	1.3 (0.8, 2.0)	39/134	1.5 (0.8, 2.7)
> 5.03–8.95	59/135	1.0 (0.6, 1.7)	29/135	1.1 (0.6, 2.1)
> 8.95	63/134	1.1 (0.6, 1.8)	39/134	1.4 (0.7, 2.7)
Dieldrin^*d*^
≤ 10.0 (LOD)	59/151	1.0	32/151	1.0
> 10.0–48.18	52/97	1.3 (0.8, 2.1)	17/97	0.7 (0.4, 1.5)
> 48.18–73.74	65/123	1.1 (0.7, 1.8)	39/123	1.2 (0.7, 2.1)
> 73.74	52/124	0.8 (0.5, 1.3)	30/124	0.8 (0.4, 1.4)
Hexachlorobenzene
≤ 159.74	49/135	1.0	23/135	1.0
> 159.74–278.998	71/134	1.4 (0.9, 2.1)	40/134	1.6 (0.9, 3.0)
> 278.998–589.30	72/134	1.4 (0.9, 2.1)	35/134	1.4 (0.7, 2.6)
> 589.30	56/135	0.9 (0.6, 1.5)	34/135	1.1 (0.6, 2.1)
Mirex^*b*^
≤ 10.0 (LOD)	133/320	1.0	68/320	1.0
> 10.0–15.47	45/97	1.1 (0.7, 1.8)	28/97	1.3 (0.8, 2.2)
> 15.47	60/98	1.5 (1.0, 2.2)	29/98	1.2 (0.7, 2.1)
^***a***^Adjusted for age, reference date year, smoking, alcohol, education, natural logarithm-transformed total lipids, and race/ethnicity. ^***b***^Lowest category consists of values < LOD. Middle and top categories, ≤ median or > median, based on observations with values among controls. ^***c***^Summed chemicals used multiple imputation data (five data sets); quartile cut points and frequencies are from first imputed data set. ^***d***^All samples with values < LOD were categorized in the lowest category. Subsequent categories were created using the 50th and 75th percentile among the distribution in controls.				

Overall, the ORs for the associations between individual OCP analytes and endometriosis that were additionally adjusted for parity and breastfeeding were generally lower than the estimates not adjusted for these factors (see Supplemental Material, Table S1). However, the association between β-HCH and ovarian endometriosis remained (third vs. lowest quartile: OR = 1.9; 95% CI: 0.9, 4.1; highest vs. lowest quartile: OR = 1.7; 95% CI: 0.7, 3.8).

With regard to serum OCP concentrations among controls with and without a history of laparoscopic evaluation, we observed a significant difference in the distribution of OCP analytes oxychlordane, *trans*-nonachlor, hexachlorobenzene, and mirex, with laparoscopic controls having systematically greater concentrations (see Supplemental Material, Table S2). We did not observe a significant difference in the distribution of OCP analytes between controls with and without a history of infertility testing (see Supplemental Material, Table S3).

## Discussion

In this population-based study, our data suggested an increased risk of endometriosis in relation to serum concentrations of β-HCH. β-HCH is not intentionally produced but is a component of technical-grade HCH, which was used as an agricultural insecticide in the United States until the mid-1970s. In addition, β-HCH is a biologically persistent unintentional by-product of γ-HCH (lindane) production [Agency for Toxic Substances and Disease Registry (ATSDR) 2005; CDC 2009]. Prior epidemiologic studies restricted to women undergoing laparoscopy have reported divergent findings for associations between endometriosis and serum or adipose tissue β-HCH concentrations, with ORs ranging from 0.77 to 2.0 (Buck Louis et al. 2012; Cooney et al. 2010; Lebel et al. 1998; Tsukino et al. 2005). In our study population, controls with a history of laparoscopy significantly differed from other controls with regard to the distribution of select OCP analytes, and had systematically larger values. Although we did not observe a statistically significant difference in the distribution of serum β-HCH concentrations between controls with and without a history of laparoscopy (*p* = 0.087), the results of our exploratory analysis generally suggest that findings based only on women undergoing laparoscopic evaluation may have been influenced by selection bias. Our results are consistent with recently published findings on a population cohort of 127 women sampled from a California telephone directory and Utah population database (Buck Louis et al. 2012). The investigators reported an adjusted OR of 1.72 (95% CI: 1.09, 2.72) for endometriosis detected by magnetic resonance imaging in relation to a 1-SD increase in the log-transformed serum concentration of β-HCH (Buck Louis et al. 2012). Similar to the population cohort in that study, the distributions of exposures in WREN controls likely represented the exposure distributions in the underlying population, because controls were randomly sampled directly from the source population from which the incident endometriosis cases emerged. Thus, the use of a population-based sampling framework in both studies may have avoided selection bias present in analyses restricted to women undergoing laparoscopy and allowed for more accurate risk estimates.

We also found the suggestion of increased endometriosis risk associated with serum concentrations of mirex, assuming that the measurement of mirex after diagnosis in cases is representative of exposure during the etiologically relevant time window. Mirex was used in the 1960s and 1970s as part of an insect control program against fire ants, with aerial application on millions of acres of southeastern U.S. states, and the chemical also was used as a fire-retardant additive. With a half-life of up to 10 years, mirex is considered one of the most stable and persistent pesticides (ATSDR 1995; Fisher 1999). In our study of U.S. Pacific Northwest health plan enrollees, only 38.2% of mirex concentration measurements were above the LOD, indicating that this potential risk factor would be unlikely to be a major contributor to endometriosis incidence in our population. However, the investigation of mirex in relation to endometriosis remains relevant because some populations may be exposed to greater concentrations of mirex, such as those who consume fish from mirex-contaminated lakes and waterways of the Great Lakes and those residing in arctic regions, particularly indigenous populations (ATSDR 1995; Kearney et al. 1999; Van Oostdam et al. 2004). One study reported the detection of mirex concentrations in the sera of Inuit populations of Greenland and Canada, ranging from 86% to 97% (Van Oostdam et al. 2004). Mirex has also been detected in the sera of 85.9% of pregnant women in an agricultural community in California (Fenster et al. 2006), suggesting that other populations also may have increased mirex exposure. The association we found between serum concentrations of mirex and endometriosis risk contrasts with the results of three small prior studies; one study of 29 cases and 51 controls reported null results (Cooney et al. 2010) and two studies reported no statistically significant difference in lipid-adjusted serum concentrations between 58 cases and 81 controls (Tsukino et al. 2005) and 86 cases and 70 controls (Lebel et al. 1998). All three of these case–control studies were conducted among laparoscopic patients. Additionally, given the small sample sizes, these studies may have been underpowered to detect an association.

In our study, the possibility existed for controls to have undiagnosed disease, because the absence of endometriosis was not confirmed surgically in controls in the parent WREN study. However, the prevalence of undiagnosed endometriosis that meets the case definition of endometriotic disease is likely to be small, possibly < 2%, which suggests that the presence of undiagnosed cases among our study controls would not substantially bias associations (Holt and Weiss 2000).

Given the number of statistical tests carried out in this study, it is possible that some of our findings were attributable to chance. It is also possible that our results were affected by misclassification of exposure. Similar to previous studies of serum and adipose tissue concentrations of OCPs and endometriosis, serum samples in our study were obtained after disease onset, a median of 1.2 years after the diagnosis date (range 6 months to 5.8 years), and serum OCP concentrations may not reflect OCP body burden at the time of disease development and progression. The timing of sample collection may have resulted in an underestimate of exposure in women who subsequently gave birth or breastfed, factors that may reduce OCP body burden (Sarcinelli et al. 2003; Soliman et al. 2003). Because parity and breastfeeding may be intermediates in the causal pathway between exposure and disease, or may be affected by endometriosis, we decided *a priori* not to adjust for these factors in the main analyses. In our exploratory analyses considering an alternative conceptual framework with parity and breastfeeding as confounders, ORs were generally lower than estimates from our main analyses that were not adjusted for these factors. However, in both sets of analyses, we observed a positive association between β-HCH and ovarian endometriosis.

## Conclusion

In this study of women enrolled in a large health care system in the U.S. Pacific Northwest, serum concentrations of β-HCH and mirex were positively associated with endometriosis. Our study suggests that exposure from extensive past use of environmentally persistent OCPs in the United States or present use in other countries may affect the health of the current generation of reproductive-age women with regard to a hormonally mediated disease.

## Supplemental Material

(348 KB) PDF
