# Supplementary material for: Organochlorine Pesticides and Risk of Endometriosis: Findings from a Population-Based Case–Control Study
Source: Environ Health Perspect. 2013 Nov 5;121(11-12):1319–24. doi: 10.1289/ehp.1306648 (PMC3855515; doi:10.1289/ehp.1306648)
Supplement: (348 KB) PDF [file ehp.1306648.s001.508.pdf]

Supplemental Material

Organochlorine Pesticides and Risk of Endometriosis: Findings from a Population-Based Case–Control Study

Kristen Upson, Anneclaire J. De Roos, Mary Lou Thompson, Sheela Sathyanarayana, Delia Scholes, Dana Boyd Barr, and Victoria L. Holt

Table of Contents

	Page
**Supplemental Material, Table S1.** Odds ratios and 95% confidence intervals for the relationship between individual organochlorine pesticides and endometriosis additionally adjusting for parity and breastfeeding, Group Health, 1996-2001.	2
**Supplemental Material, Table S2.** Distribution of serum organochlorine pesticides among controls by history of laparoscopy, Group Health, 1996-2001.	4
**Supplemental Material, Table S3.** Distribution of serum organochlorine pesticides among controls by history of infertility testing, Group Health, 1996-2001.	5

Supplemental Material, Table S1. Odds ratios and 95% confidence intervals for the relationship between individual organochlorine pesticides and endometriosis additionally adjusting for parity and breastfeeding, Group Health, 1996-2001.

Pesticide	All endometriosis		Ovarian endometriosis	
Quartiles (pg/g serum)	Case/control (n)	aOR[a] (95% CI)	Case/Control (n)	aOR[a] (95% CI)
β-HCH				
≤26.99	49/126	1.0	17/126	1.0
>26.99-43.06	41/127	0.7 (0.4, 1.3)	21/127	1.0 (0.5, 2.2)
>43.06-74.01	78/127	1.3 (0.8, 2.3)	42/127	1.9 (0.9, 4.1)
>74.01	64/127	1.0 (0.5, 1.8)	42/127	1.7 (0.7, 3.8)
γ-HCH[b]				
≤10.0 (LOD)	129/295	1.0	69/295	1.0
>10.0-13.89	54/112	1.1 (0.8, 1.7)	29/112	1.1 (0.6, 1.9)
>13.89	61/112	1.3 (0.9, 1.9)	32/112	1.2 (0.7, 2.0)
∑HCH (mol/g serum)[c]				
≤0.12	54/134	1.0	24/134	1.0
>0.12-0.18	48/135	0.8 (0.5, 1.2)	21/135	0.8 (0.4, 1.7)
>0.18-0.29	79/134	1.2 (0.7, 1.9)	45/134	1.5 (0.8, 2.9)
>0.29	67/135	0.8 (0.5, 1.4)	42/135	1.1 (0.6, 2.3)
Heptachlor epoxide				
≤17.10	40/110	1.0	21/110	1.0
>17.10-26.19	55/110	1.5 (0.9, 2.5)	22/110	1.0 (0.5, 2.1)
>26.19-42.91	59/110	1.3 (0.7, 2.2)	34/110	1.2 (0.6, 2.3)
>42.91	51/109	1.0 (0.5, 1.8)	28/109	0.8 (0.4, 1.8)
Oxychlordane				
≤28.31	52/124	1.0	24/124	1.0
>28.31-51.96	46/125	0.7 (0.4, 1.2)	23/125	0.7 (0.4, 1.5)
>51.96-79.55	57/126	0.8 (0.5, 1.4)	34/126	1.0 (0.5, 1.9)
>79.55	73/124	0.8 (0.5, 1.5)	45/124	0.9 (0.5, 2.0)
trans-nonachlor				
≤51.37	55/134	1.0	21/134	1.0
>51.37-75.15	55/135	0.9 (0.6, 1.5)	31/135	1.2 (0.6, 2.4)
>75.15-107.65	56/134	0.9 (0.5, 1.4)	33/134	1.1 (0.5, 2.2)
>107.65	82/135	1.1 (0.6, 1.9)	47/135	1.3 (0.6, 2.8)
∑Chlordane (mol/g serum)[c]				
≤0.21	53/134	1.0	23/134	1.0
>0.21-0.33	56/135	1.0 (0.6, 1.7)	24/135	1.0 (0.5, 1.9)
>0.33-0.52	57/134	1.0 (0.6, 1.6)	36/134	1.3 (0.6, 2.5)
>0.52	82/135	1.1 (0.7, 2.0)	49/135	1.3 (0.6, 2.8)

Pesticide	All endometriosis		Ovarian endometriosis	
Quartiles (pg/g serum)	Case/control (n)	aOR[a] (95% CI)	Case/Control (n)	aOR[a] (95% CI)
p,p'-DDE				
≤905.86	55/134	1.0	25/134	1.0
>905.86-1575.51	69/134	1.1 (0.7, 1.8)	38/134	1.2 (0.7, 2.3)
>1575.51-2816.55	62/134	0.9 (0.5, 1.4)	31/134	0.8 (0.4, 1.6)
>2816.55	61/135	0.8 (0.5, 1.3)	37/135	0.9 (0.4, 1.8)
p,p'-DDT				
≤18.79	58/128	1.0	21/128	1.0
>18.79-28.36	58/128	0.8 (0.5, 1.4)	33/128	1.2 (0.6, 2.3)
>28.36-44.38	55/128	0.7 (0.4, 1.1)	32/128	1.0 (0.5, 1.9)
>44.38	66/129	0.8 (0.4, 1.3)	38/129	1.0 (0.5, 2.0)
∑DDT (mol/g serum)[c]				
≤2.88	55/135	1.0	25/135	1.0
>2.88-5.03	71/134	1.2 (0.8, 1.9)	39/134	1.3 (0.7, 2.4)
>5.03-8.95	59/135	0.8 (0.5, 1.4)	29/135	0.8 (0.4, 1.6)
>8.95	63/134	0.9 (0.5, 1.5)	39/134	1.0 (0.5, 2.1)
Dieldrin[d]				
≤10.0 (LOD)	59/151	1.0	32/151	1.0
>10.0-48.18	52/97	1.2 (0.8, 2.0)	17/97	0.7 (0.3, 1.4)
>48.18-73.74	65/123	1.1 (0.7, 1.7)	39/123	1.0 (0.6, 1.9)
>73.74	52/124	0.7 (0.4, 1.2)	30/124	0.6 (0.3, 1.1)
Hexachlorobenzene				
≤159.74	49/135	1.0	23/135	1.0
>159.74-278.998	71/134	1.3 (0.8, 2.1)	40/134	1.5 (0.8, 2.8)
>278.998-589.30	72/134	1.3 (0.8, 2.0)	35/134	1.3 (0.7, 2.4)
>589.30	56/135	0.9 (0.5, 1.5)	34/135	1.0 (0.5, 1.8)
Mirex[b]				
≤10.0 (LOD)	133/320	1.0	68/320	1.0
>10.0-15.47	45/97	1.1 (0.7, 1.7)	28/97	1.3 (0.7, 2.2)
>15.47	60/98	1.3 (0.9, 2.0)	29/98	1.0 (0.6, 1.8)

Abbreviations: OR=Odds ratio; 95% CI=95% confidence interval; LOD=limit of detection; HCH=hexachlorocyclohexane; DDE=dichlorodiphenyldichloroethylene; DDT=dichlorodiphenyltrichloroethane. [a]Odds ratio adjusted for age, reference date year, smoking, alcohol, education, natural logarithm transformed total lipids, race/ethnicity, and a composite variable for parity and breastfeeding. [b]Lowest category consists of values <LOD. Middle and top categories, ≤median or >median, based on observations with values among controls. [c]Summed chemicals used multiple imputation data (five datasets); Quartile cutpoints and frequencies from first imputed dataset. [d]All samples with values below the level of detection were categorized in the lowest category. Subsequent categories were created using the 50th and 75th percentile among the distribution in controls.

Supplemental Material, Table S2. Distribution of serum organochlorine pesticides among controls by history of laparoscopy, Group Health, 1996-2001.

Pesticide (pg/g serum)	Past laparoscopy[a] (n=47) Median (IQR)	No past laparoscopy (n=491) Median (IQR)	*P*-value[b]
β-HCH	47.58 (38.27, 98.72)	42.74 (26.17, 73.40)	0.087
γ-HCH	<10.00 (<10.00, 15.13)	<10.00 (<10.00, 13.25)	0.153
Heptachlor epoxide	25.69 (19.59, 41.46)	26.38 (16.75, 43.75)	0.952
Oxychlordane	62.40 (43.23, 99.66)	50.95 (25.62, 77.79)	0.035
trans-nonachlor	81.14 (64.14, 131.97)	73.66 (49.95, 107.35)	0.045
p,p'-DDE	1751.52 (825.69, 3798.72)	1563.26 (909.65, 2776.09)	0.389
p,p'-DDT	27.43 (17.49, 54.86)	28.37 (18.89, 43.83)	0.927
Dieldrin	55.88 (<10.00, 77.96)	46.76 (<10.00, 72.86)	0.426
Hexachlorobenzene	387.85 (237.84, 742.02)	268.66 (156.81, 586.09)	0.019
Mirex	10.96 (<10.00, 16.71)	<10.00 (<10.00, 12.79)	0.020

Abbreviations: IQR=Interquartile range; HCH=hexachlorocyclohexane; DDE=dichlorodiphenyldichloroethylene; DDT=dichlorodiphenyltrichloroethane.

[a]History of laparoscopy was based on self-report of laparoscopy before the reference date.

[b]*P*-value from Wilcoxon rank sum test testing whether the distributions of OCP analyte concentrations are systematically different between controls with and without a history of laparoscopy. For this analysis, serum measurements less than the limit of detection (LOD) were substituted with the value of the limit of detection divided by the square root of two (LOD/√2).

Supplemental Material, Table S3. Distribution of serum organochlorine pesticides among controls by history of infertility testing, Group Health, 1996-2001.

Pesticide (pg/g serum)	Infertility testing history[a] (n=39) Median (IQR)	No infertility testing history (n=499) Median (IQR)	*P*-value[b]
β-HCH	49.33 (34.51, 83.61)	42.79 (26.93, 73.72)	0.263
γ-HCH	10.74 (<10.00, 14.20)	<10.00 (<10.00, 13.29)	0.128
Heptachlor epoxide	23.99 (16.15, 41.37)	26.38 (17.10, 43.23)	0.766
Oxychlordane	54.25 (38.85, 81.49)	51.15 (27.09, 78.84)	0.402
trans-nonachlor	81.24 (69.09, 116.09)	73.66 (50.86, 107.57)	0.157
p,p'-DDE	1732.02 (940.30, 2901.02)	1574.52 (900.88, 2816.55)	0.634
p,p'-DDT	28.86 (17.51, 51.19)	28.30 (19.04, 44.02)	0.859
Dieldrin	50.59 (<10.00, 75.05)	48.15 (<10.00, 73.67)	0.727
Hexachlorobenzene	350.13 (179.59, 685.24)	277.99 (159.26, 586.45)	0.361
Mirex	<10.00 (<10.00, 17.44)	<10.00 (<10.00, 12.79)	0.088

Abbreviations: IQR=Interquartile range; HCH=hexachlorocyclohexane; DDE=dichlorodiphenyldichloroethylene; DDT=dichlorodiphenyltrichloroethane.

[a]History of infertility was based on self-report of having tests done for infertility.

[b]*P*-value from Wilcoxon rank sum test testing whether the distributions of OCP analyte concentrations are systematically different between controls with and without a history of infertility testing. For this analysis, serum measurements less than the limit of detection (LOD) were substituted with the value of the limit of detection divided by the square root of two (LOD/√2).
